# Is fibrin sealant effective and safe in total knee arthroplasty? A meta-analysis of randomized trials

**DOI:** 10.1186/1749-799X-9-36

**Published:** 2014-05-16

**Authors:** Hongsheng Wang, Liancheng Shan, Hui Zeng, Mengxiong Sun, Yingqi Hua, Zhengdong Cai

**Affiliations:** 1 Department of Orthopedics, Tenth People’s Hospital of Tongji University, 301 Yanchang Road, Shanghai 200072, China; 2 Postdoctoral Research Station of Biomedical Engineering, School of Life Science and Technology, Tongji University, Shanghai 200092, China; 3 Department of Orthopedics, Advanced Institute of Translation Medicine, Tongji University, Shanghai 200092, China

**Keywords:** Total knee arthroplasty (TKA), Fibrin sealant, Meta-analysis, Efficacy and safety

## Abstract

The objective of this study was to evaluate the efficacy and safety of fibrin sealant in patients following total knee arthroplasty (TKA). A comprehensive literature search of the electronic databases PubMed, MEDLINE, Web of Science, and Cochrane Library for published randomized controlled trials (RCTs) was undertaken. The evidence base was critically appraised using a tool from the Cochrane Bone, Joint and Muscle Trauma Group. Eight RCTs involving 641 patients were included. The use of fibrin sealant significantly reduced postoperative drainage (weighted mean difference (WMD) −346, 95% confidence interval (CI) −496.29 to −197.54, *P* < 0.00001) and blood transfusions (risk ratio (RR) 0.47, 95% CI 0.35 to 0.63, *P* < 0.00001) and led to a significant improvement in the range of motion (WMD 16.59, 95% CI 6.92 to 26.25, *P* = 0.0008). However, using fibrin sealant did not significantly reduced total blood loss (WMD −305.25, 95% CI −679.44 to 68.95, *P* = 0.11). Regarding complications, there were no significant differences in any adverse events, fever, infection, or hematoma among the study groups. In conclusion, the present meta-analysis indicates that the use of fibrin sealant was effective and safe as a hemostatic therapy for patients with TKA.

## Introduction

Total knee arthroplasty (TKA) is a safe and effective treatment for alleviation of pain and restoration of function for end-stage knee diseases. However, TKA is associated with significant blood loss because of extensive dissections through bony and fibrotic tissue [1,2]. Blood loss is a common problem during and after TKA, which can result in the need for transfusions, postoperative anemia, and increased costs for health care [3]. In addition, blood transfusions may also increase the patient’s risk for postoperative infection [4]. Various technologies have been employed to minimize the need for allogeneic blood transfusion. Perioperative strategies include the use of autologous blood donation and administration of erythropoietin [5,6]; intraoperative measures include acute normovolemic hemodilution, hypotensive anesthesia, intraoperative blood salvage, specialized cautery, topical hemostatic agents, and pharmacologic agents [7].

The use of fibrin sealant (i.e., fibrin glues, fibrin tissue adhesives) could be a promising approach to reduce bleeding and consequently lead to lower transfusion rates [8]. Fibrin sealants have been increasingly used as adjunctive surgical and hemostatic agents for over 20 years [9]. A number of systematic reviews have examined the efficacy and safety in cardiac surgery [10], thoracic surgery [11], plastic and reconstructive surgery [12], and orthopedic surgery settings [13]. The main components of the sealant are fibrinogen, factor XIII, thrombin, and antifibrinolytic agents, such as aprotinin or tranexamic acid. Fibrin sealants achieve their local hemostatic effects by reproducing the last step of the coagulation cascade, thereby facilitating formation of a stable fibrin clot and subsequent hemostasis [14].

To date, several randomized controlled trials (RCTs) have been done to examine the efficacy of fibrin sealants for TKA. Results from these trials have indicated that fibrin sealants were efficacious and promising agents in TKA. However, at least two studies found that fibrin sealants had no effect on drain output or functional recovery following TKA [15,16]. The aim of this review is to evaluate the effectiveness of fibrin sealant treatment in reducing postoperative blood loss and transfusion for patients undergoing TKA.

## Method and material

### Literature search

A systematic search of literature was conducted on PubMed, Web of Science, MEDLINE, and Cochrane Library databases for relevant articles published until 27 June 2013. Articles were identified using the following search terms: ‘fibrin sealant’ OR ‘fibrin glues’ OR ‘fibrin tissue adhesives’ AND ‘total knee arthroplasty’ OR ‘total knee replacement’. The language of the publications was limited to English. The title and abstract of studies identified in the search were scanned to exclude any clearly irrelevant studies. Furthermore, references of identified articles were also searched manually. A search for unpublished literature was not performed.

### Study selection

To minimize any possible selection bias, the following criteria were used: (*i*) the study should be a randomized controlled trial, (*ii*) the study should compare the use of fibrin sealant (fibrin glue, fibrin tissue adhesive) with no sealant or placebo or standard method of hemostasis or other hemostatic agents, and (*iii*) the study should report the clinical outcome of the fibrin sealant in TKA. We excluded articles of studies on animals, reviews, editorial letters, case reports, poster sessions, or studies with insufficient data. Firstly, we screened the title and abstract to see whether they met the inclusion criteria. Then, based on the initial screening, we scrutinized the full manuscript of studies that needed further examination.

### Study quality

In order to assess the methodological quality of included studies, two authors used a modification of the generic evaluation tool used by the Cochrane Bone, Joint and Muscle Trauma Group [17]. The methodological quality of each trial was scored and ranged from 0 to 24. Any controversy was cross-checked and resolved by a third author to reach a final consensus.

### Date extraction

Two authors extracted independently all relevant data in the specially predesigned data form; any disagreements during the extraction process were resolved by consulting the first author until a final consensus was achieved. For each trial, the following data were collected: the author details; year of publication; country in which the study was performed; study design; general characteristics of patients, interventions, and control treatment; outcome data; and definitions of outcomes from studies.

### Outcome measures

The primary outcome measures included in this review were postoperative drainage, total blood loss, blood transfusion, hemoglobin (Hb) drop, range of motion, and length of hospital stay. Secondary outcome measures were data about safety, including any adverse events (AEs), fever, infection, and hematoma.

### Statistical analysis

Statistical analysis was performed with Review Manager version 5.1. For dichotomous data, the risk ratio (RR) and 95% confidence interval (CI) were calculated. Weighted mean difference (WMD) was used to report continuous outcomes. Clinical heterogeneity was evaluated by considering the design of each trial. If clinical heterogeneity did not exist, the statistical heterogeneity was assessed using the chi-square test (*P* < 0.1 indicating significance) and quantified using the *I*^2^ statistic (*I*^2^ value > 50% means significant heterogeneity). When there was a significant heterogeneity across the included trials, a random effects model was used for analysis. If no heterogeneity was present among studies, the fixed effects model was performed. Funnel plot was performed to identify the possibility of publication bias.

## Results

### Study characteristics and quality assessment

**Figure 1:**
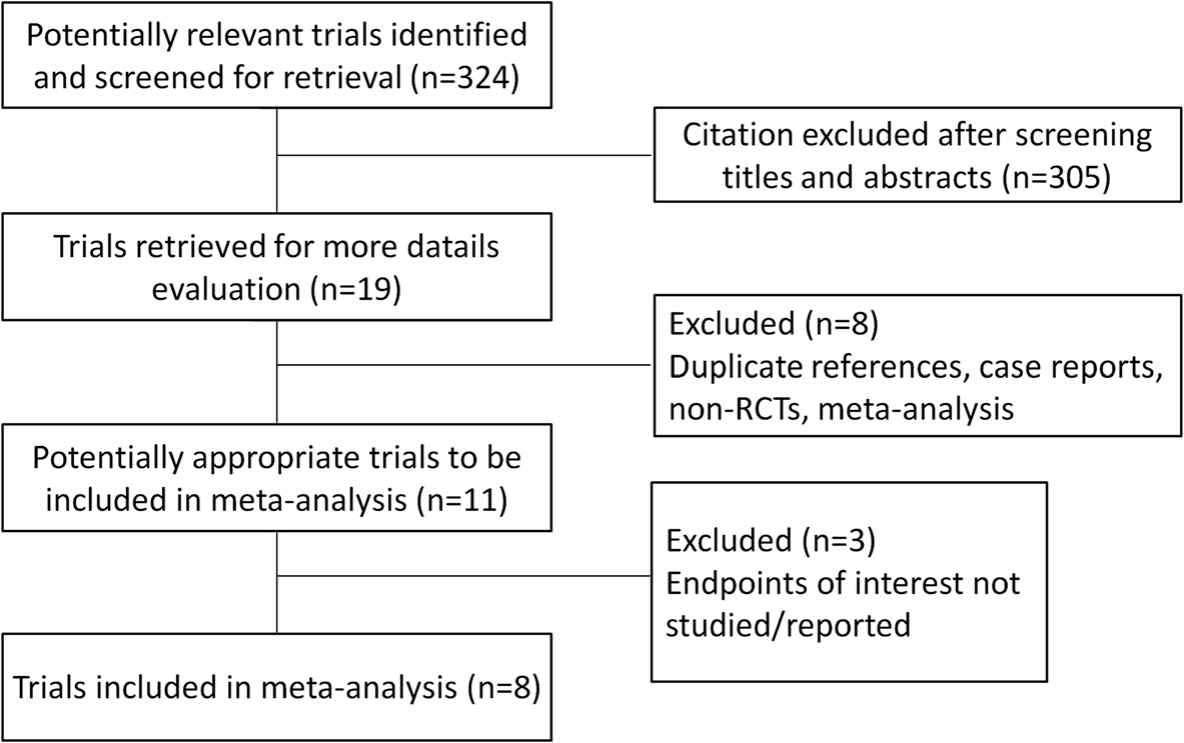
Process of study selection of randomized controlled trials.

The detailed steps of our literature search are shown in Figure 1. Briefly, an initial search revealed 324 potentially relevant studies of which eight RCTs fully met the inclusion criteria [15,16,18-23]. The major characteristics of the included studies are outlined in Table 1. A total of 641 participants were enrolled in the eight studies, with sample sizes ranging from 24 to 198 patients. There were 303 patients in the fibrin sealant group and 338 patients in the control group. In the included studies, participants had similar demographic data in the two groups. The volume of fibrin sealant applied to the surgical site varied from 2 to 20 ml, and Notarnicola et al. [21] assessed the efficacy of two different doses of fibrin sealant (5 and 10 ml) in TKA. The use of antifibrinolytic agents also varied with the different agents studied. The total quality assessment score (QAS) was generally moderate. The mode was 24 (the highest possible score) and the range was 14 to 21, with only one study scoring > 20.

**Table 1:** Characteristics of randomized controlled trials included in the meta-analysis

**Study**	**Study design**	**Country, year**	**QAS**	**Interventions**	**Number of patients (FS/control)**	**Outcomes**
Heyse et al. [15]	Double-blind, RCT	USA, 2013	21	Fibrin sealant 10 ml before tourniquet release	98/100	TBL: FS 1,441 ml *vs.* control 1,409 ml
				Control: no fibrin sealant		24 h postoperative blood drainage: FS 780 ml *vs.* control 673 ml
						48 h postoperative Hb drop: FS 3.47 g/l *vs.* control 3.84 g/l
Kluba et al. [19]	Single-center, prospective, RCT	Germany, 2012	17	Fibrin sealant 2 ml before closing the wound	12/12	Postoperative blood drainage: FS 475.4 ml *vs.* control 813.3 ml
				Control: no fibrin sealant		8 days postoperative ROM: FS 90.8 *vs.* control 85.4
						Length of hospital stay: FS 12.67 days *vs.* control 13.67 days
Levy et al. [18]	Multicenter, prospective, RCT	Israel, 1999	18	Fibrin sealant 10–20 ml before closing the wound	29/29	TBL: FS 1,063 ml *vs.* control 1,768 ml
				Control: no fibrin sealant		Postoperative blood loss: FS 360 ml *vs.* control 878 ml
						Postoperative Hb drop: FS 25 g/l *vs.* control 37 g/l
						Blood transfusion rate: FS 5 patients *vs.* control 16 patients
Molloy et al. [20]	Prospective, RCT	Northern Ireland, 2007	14	Fibrin sealant 6 ml before the prosthesis is inserted, then 4 ml after placement of the prosthesis	50/50	TBL: FS 1,190 ml vs. control 1,415 ml
				Control: no pharmacological intervention		Postoperative Hb drop: FS 2.68 g/dl vs. control 3.20 g/dl
						Blood transfusion rate: FS 7 patients *vs.* control 11 patients
Notarnicola et al. [21]	Prospective, RCT	Italy, 2012	15	Fibrin sealant (5 and 10 ml) before closing the wound and releasing the tourniquet	30/30/30	3 days postoperative blood drainage: FS (5 ml) 415 ml *vs.* FS (10 ml) 228.3 ml *vs.* control 815 ml
				Control: no fibrin sealant		Postoperative Hb drop: FS (5 ml) 2.6 mg/dl *vs.* FS (10 ml) 2.5 mg/dl *vs.* control 3.7 mg/dl
						Blood transfusion rate: FS (5 ml) 10 patients *vs.* FS (10 ml) 7 patients *vs.* control 19 patients
						7 days postoperative ROM: FS (5 ml) 96.5 *vs.* FS (10 ml) 98.8 *vs.* control 75.5
						Length of hospital stay: FS (5 ml) 10 days *vs.* FS (10 ml) 9.2 days *vs.* control 13.6 days
Sabatini et al. [22]	Prospective, RCT	Italy, 2012	18	Fibrin sealant 5 ml before the prosthesis is inserted	35/35	3 days postoperative blood drainage: FS 910 ml *vs.* control 1,250 ml
				Control: no fibrin sealant		24 h postoperative Hb drop: FS 2.6 g/dl *vs.* control 3 g/dl
						Blood transfusion rate: FS 5 patients *vs.* control 15 patients
Skovgaard et al. [16]	Double-blind, prospective, RCT	Denmark, 2013	21	Fibrin sealant 10 ml after the prosthesis is inserted	24/24	24 h postoperative blood drainage: FS 582 ml *vs.* control 576 ml
				Control: placebo		7 days postoperative ROM: FS 88 *vs.* control 86
Wang et al. [23]	Multicenter, prospective, RCT	USA, 2001	16	Fibrin sealant 10 ml before closing the wound and releasing the tourniquet	25/28	12 h postoperative blood drainage: FS 184.5 ml *vs.* control 408.3
						24 h postoperative Hb drop: FS 20.1 g/l *vs.* control 27.3 g/l
				Control: no placebo		
						Blood transfusion rate: FS 9 patients *vs.* control 14 patients

*RCT* randomized controlled trial, *FS* fibrin sealant, *QAS* quality assessment score, *Hb drop* hemoglobin drop, *TBL* total blood loss, *ROM* range of motion.

### Meta-analysis of efficacy

#### Blood loss

**Figure 2:**
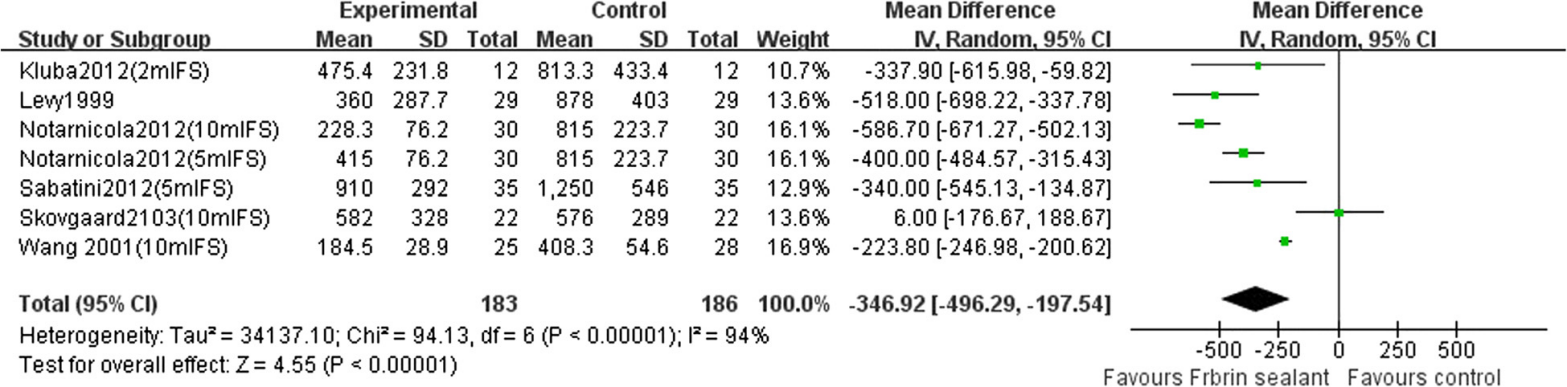
Forest plot of combined WMD values for postoperative drainage in TKA.

**Figure 3:**
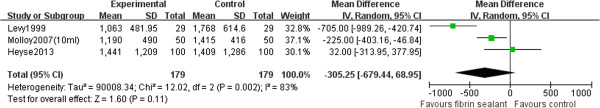
Forest plot of combined WMD values for total blood loss in TKA.

Information on blood loss was available for all the studies included in the meta-analysis. The studies measure external blood loss as the volume contained in the drain, total blood loss, or a combination of both. Six studies [16,18,19,21-23] (369 patients) reported postoperative drainage. Postoperative time until the evaluation of the drainage was different in each trial: 12 h for the study by Wang et al., 24 h for the study by Skovgaard et al., 72 h in the studies by Notarnicola et al. and Sabatini et al., and no time given in the study by Levy et al. The general appraisal of the postoperative drainage indicated that the use of fibrin sealant significantly reduced postoperative drainage (WMD −346, 95% CI −496.29 to −197.54, *P* < 0.00001, Figure 2). There was, however, significant heterogeneity between studies (*P* < 0.001, *I*^*2*^ = 94%). Three studies [15,18,20] (358 patients) reported total blood loss (TBL). As shown in Figure 3, our meta-analysis showed that using fibrin sealant did not lead to a significant reduction of total blood loss (WMD −305.25, 95% CI −679.44 to 68.95, *P* = 0.11, Figure 3). Again, there was significant heterogeneity between studies (*P* = 0.002, *I*^*2*^ = 83%).

#### Hemoglobin drop

**Figure 4:**
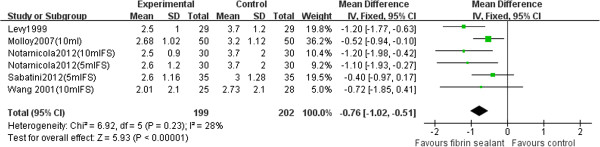
Forest plot of combined WMD values for Hb drop in TKA.

When the result variable was the decrease in Hb level after the operation, five studies [18,20-23] (401 patients) were used for the meta-analysis. Postoperative time to evaluate treatment was 24 h for the Wang et al. study and Sabatini et al. study and was not given by the studies of Notarnicola et al., Levy et al., and Molloy et al. The general estimation for Hb level reduction indicated that the use of fibrin sealant significantly decreased blood loss (WMD −0.76, 95% CI −1.02 to −0.51, *P* < 0.00001, Figure 4). There was no significant heterogeneity between studies (*P* = 0.23, *I*^*2*^ = 28%).

#### Blood transfusion

**Figure 5:**
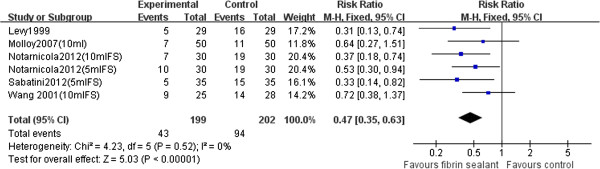
Forest plot of combined RR values for the number of patients requiring blood transfusions in TKA.

Five studies [18,20-23] (401 patients) were used to perform the meta-analysis with the blood transfusion. As shown in Figure 5, our pooled results showed that the blood transfusion requirements in the fibrin sealant group were significantly lower than those in the control group (RR 0.47, 95% CI 0.35 to 0.63, *P* < 0.00001, Figure 5). There was no significant heterogeneity between studies (*P* = 0.52, *I*^2^ = 0%).

#### Range of motion

**Figure 6:**
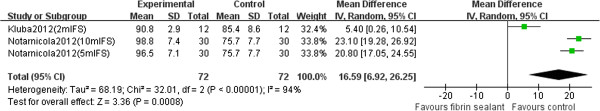
Forest plot of combined WMD values for range of motion in TKA.

Two studies [19,21] (144 patients) were used to perform the meta-analysis with the range of motion. Postoperative time to evaluate treatment was 8 days for the Kluba et al. study and 17 days for the Notarnicola et al. study. As shown in Figure 6, our pooled results showed that the range of motion in the fibrin sealant group was significantly better than that in the control group (WMD 16.59, 95% CI 6.92 to 26.25, *P* = 0.008, Figure 6). There was significant heterogeneity between studies (*P* < 0.001, *I*^*2*^ = 94%).

#### Length of hospital stay

**Figure 7:**
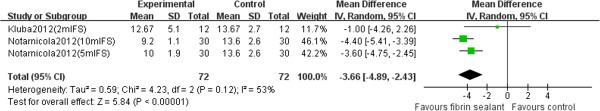
Forest plot of combined WMD values for length of hospital stay in TKA.

Two studies [19,21] (144 patients) reported the length of hospital stay, and the meta-analysis results showed that the use of fibrin sealant was significantly associated with a reduced hospital stay (WMD −3.66, 95% CI −4.89 to −2.43, *P* < 0.00001, Figure 7). There was significant heterogeneity between studies (*P* = 0.12, *I*^*2*^ = 53%).

### Meta-analysis of safety

Results concerning safety are demonstrated in Table 2. Data regarding occurrence of any adverse events, fever, infection, and hematoma are extracted from each study. The results of the meta-analysis showed that the incidences of adverse events, fever, infection, and hematoma were similar between the two groups. However, the number of events was too small to draw firm conclusions. We did not evaluate the risk of deep venous thrombosis because of insufficient data for pooling.

**Table 2:** Summary of adverse events in randomized controlled trials included in the meta-analysis

**Outcome**	**Studies**	**Fibrin sealant events/total**	**Control events/total**	**Heterogeneity (*P, I***^***2***^**)**	**Statistical method**	**Effect estimate**	***P *** **value**
Any adverse events	3	61/164	50/164	0.007, 80%	Risk ratio (M-H, fixed, 95% CI)	1.21 (0.63, 2.31)	0.57
Fever	3	34/164	32/164	0.94, 0%	Risk ratio (M-H, fixed, 95% CI)	1.06 (0.79, 1.44)	0.69
Infection	2	3/129	2/129	0.71, 0%	Risk ratio (M-H, fixed, 95% CI)	1.50 (0.26, 8.72)	0.65
Hematoma	2	3/60	10/63	0.90, 0%	Risk ratio (M-H, fixed, 95% CI)	0.31 (0.09, 1.08)	0.07

*M-H* Mantel-Haenszel.

## Discussion

The benefit of using fibrin sealant has been demonstrated in several clinical studies, most of which show reduced blood loss, lower transfusion rate, improved range of motion, and shorter hospital stay. Similarly, the results of our meta-analysis demonstrated a statistically significant benefit in using fibrin sealant in patients following TKA as well. However, these results must be interpreted with caution because of the significant heterogeneity in trial findings.

Most of the observed heterogeneity seemed to be from clinical practice or trial methodology. First, for time of evaluation, the outcome measures were different. To avoid a possible bias, the time of evaluation period was considered ‘short term’ (ranging 12 to 72 h). For example, drainage output at 24 h in the Skovgaard et al. study was around 580 ml, as compared to 878 ml in the Levy et al. study [18] and 408 ml in the Wang et al. study [23] after 12 h. Besides, the amount of fibrin sealant used may influence the blood drainage: Levy et al. [18] used 10–20 ml, Kluba et al. [19] used 2 ml, and Notarnicola et al. [21] assessed the efficacy of two different doses (5 and 10 ml). However, the dose of fibrin sealant did not appear to have any bearing on the results. Furthermore, tourniquet time and tourniquet pressure should be kept short and low, and differences in these trials may also influence the outcome. Also, fibrin sealant may contain tranexamic acid, which has been demonstrated to be effective on its own in reducing blood loss. Finally, the control group in this review consisted of a variety of interventions including no fibrin sealant, placebo, and standard method of treatments, resulting in clinical and methodological heterogeneity. Subgroup analysis by the evaluation time and dose of fibrin sealant was constrained by the small number of trials.

Bleeding remains an ongoing concern after TKA. Fibrin sealants are topically applied hemostatic agents that reduce the time required to achieve hemostasis as well as the volume of blood loss. Wang et al. [23] found that there was a statistically significant reduction in blood loss 12 h postoperatively (184.5 ml in the fibrin sealant group *vs.* 408.3 ml in the control group), as well as a minor Hb reduction on the first postoperative day as compared with baseline (14.9% *vs.* 20.7%). However, in the Heyse et al. study [15], it was found that the use of topical fibrinogen did not lead to a relevant reduction in blood loss or transfusions in primary TKA. They considered that suction drainage may have affected the impact of topically applied fibrinogen. Molloy et al. [20] reported that the main cause was they did not spray the prepared bone surfaces before placement of the cementless components. Additionally, the amount of blood loss after TKA is often underestimated because measuring blood loss is a complex science in a controlled clinical trial environment. This may be one reason why the use of fibrin sealant did not significantly reduce total blood loss compared with the control group.

In the study by Kluba et al. [19], fibrin sealant patients showed a slightly better range of motion and functional knee recovery compared with the control group of patients with a maximum flexion 8 days after surgery, but the difference missed statistical significance (*P* = 0.052). Similarly, Heyse et al. [15] had shown that there was no difference between the two groups at 6 weeks follow-up. However, in the Notarnicola et al. [21] study, functional recovery was quicker in patients administered the fibrin sealant. They believed that fibrin sealant use allowed an immediate control of bleeding, which may have a beneficial effect on the achievement of early and better postoperative rehabilitation. The pooled data showed that the difference was not significant between the groups. However, it is important to state that the range of motion include only a few trials (less than 2) and have a small sample size (less than 144). Further randomized prospective studies will be needed to clarify the relationship between the use of fibrin sealant in TKA and the functional results.

Postoperative thromboembolism is one of the most serious and potentially fatal complications after TKA [24]. The Italian Agency of Drugs (AIFA) had informed that the use of a spray device to apply fibrin sealant can produce massive embolism. They recommend to use a spray device with pressure of less than 2.0–2.5 bar, to apply fibrin sealant (Quixil, a commercial fibrin sealant; OMRIX Biopharmaceuticals, Ltd., Kiryat-Ono, Israel) from a minimal distance of 10–15 cm, and to monitor patient spray application. Sabatini et al. [22] did not find any clinical signs of pulmonary embolism with application of these recommendations. Levy et al. [18] believed that the use of fibrin sealant may allow the use of full-dose preoperative thromboprophylaxis with low molecular weight heparin, thereby reducing the risk of deep vein thrombosis without increasing the risk of postoperative bleeding in patients following TKA. Additionally, our pooled results showed that there were no significant differences on complications in the fibrin sealant compared with the control group, including AEs, infection, fever, and hematoma.

The cost of fibrin sealant also warrants discussion. The beneficial effects of using fibrin sealant are associated with reduced blood loss and decreased rates of allogeneic red blood cell (RBC) transfusion [13]. In the study by Molloy et al. [20], the cost of the pharmaceutical intervention involved in the topical fibrin group was £380, which did not justify its additional cost. However, in the trial by Kluba et al. [19], results showed that in daily clinical practice, a routine application of fibrin sealant adds costs to TKA treatment without reducing the number of transfused RBC. Steuten et al. [25] performed a simple cost model to compare the cost of using a commercial fibrin sealant. Their conclusion is that whether using fibrin sealant is cost saving strongly depends on the amount used, the achieved reduction in hospital length of stay, and the price of fibrin sealant.

There are several limitations to our meta-analysis. First, we only included the study in English, and some relevant studies might not be included in the review. The few numbers of studies included in our analysis would limit the statistical power. Additionally, most of the studies did not report the results of all outcomes selected; each outcome measure was assessed in a variable number of studies and in different studies which could make comparison difficult between different domains. Furthermore, the methodological quality of the trials was poor, with only two trials judged to be double-blind. Nevertheless, our meta-analysis has several strengths. First, this review combined relevant literature published up to June 2013, with eight RCTs included in our meta-analysis. Furthermore, the present study is the first systematic review assessing the efficacy and safety of fibrin sealant treatment in patients with TKA.

## Conclusions

This meta-analysis investigated the efficacy and safety of fibrin sealant in terms of blood loss, Hb level, range of motion, length of hospital stay, and complications. Based on our findings, the use of fibrin sealant was effective and safe as a hemostatic therapy for patients with TKA. Given the limitation in this study, fibrin sealant treatment should be carefully considered for each patient. Further well-designed and large-scale clinical trials and systemic reviews are required to confirm these findings.

## Competing interests

The authors declare that they have no competing interests.

## Authors’ contributions

HW and LS conceived and designed the study. ZC provided the financial support. YH and ZC provided the study materials or patients. HW and MS collected and assembled the data. HZ and LS analyzed and interpreted the data. All authors wrote the manuscript. All authors read and approved the final manuscript.

## Authors’ information

HW and LS are co-first authors.
